# Addressing health systems strengthening through an health equity lens

**DOI:** 10.1186/1472-6963-13-S2-S13

**Published:** 2013-05-31

**Authors:** Mickey Chopra

**Affiliations:** 1 Chief of Health, UNICEF, NY, USA

## 

Addressing inequalities in health outcomes especially for women and children is, perhaps, the most important challenge towards achieving sustainable health gains. Despite impressive improvements in overall indicators of health over recent decades, health inequalities within and between countries persist and, in many cases, have widened and continue to widen. At the global level, the survival gap between poor and rich children has been growing. For example, a child born in Sub-Saharan Africa in 1970 faced a risk of dying before his or her fifth birthday that was nine times greater than a child born in an industrialized country. In 1990, the base year of the Millennium Development Goals (MDGs), the same risk was 19 times greater; in 2006, it was 27 times greater. A new global movement called A Promise Renewed aims to work with countries to end all preventable child deaths and reduce the widening inequalities between countries.

Inequalities between poor and rich children within developing countries are responsible for half or more of the global under-5 mortality gap. On average, children in the poorest 20% of a developing country population are about twice as likely to die or to be malnourished as those in the better-off 20%. If the death rates of all children in developing countries could be reduced to the level currently prevailing in the best-off child population of those countries, the number of under-5 deaths could be reduced by half or more.

There is ample evidence that packages of simple, highly cost-effective interventions have the potential to prevent the majority of maternal, newborn, and child deaths. Attaining universal coverage, especially for essential high-impact interventions for maternal and child health, is therefore rightly being promoted as a global health priority. However, whilst there is a consensus on the goal of universal coverage, divergent paths are being taken — leading to very different outcomes. Many countries have generally focused upon a geographical expansion of a facility-based health care delivery model, with the aim of achieving universality. The success of this strategy has depended upon significant donor and domestic investment in a primary health care approach and, when applied consistently, has succeeded in expanding accessibility to basic maternal, child, and other MDG-related health services. But the relative high costs and difficulty in deploying and retaining skilled health professionals to serve marginalized populations has meant that large segments of the population, mostly very low-income families in urban slums and those living in rural areas, remain beyond the reach of the conventional facility-based model.

As new and more sophisticated interventions are introduced they are being captured predominantly by those served with existing health care infrastructure, and widening inequalities. Of the 24 countries making the greatest reductions in under-5 mortality, 16 have widened disparities in mortality between the richest and poorest during the same time.

Experience and evidence clearly showing that a “business as usual” approach with a focus on secondary and tertiary services in urban areas will result, at best, in reaching the MDGs by vastly increasing inequalities, as the elites rapidly capture both public and private health and social welfare resources. One analysis of expenditures in Guinea, Ghana, and Côte d’Ivoire, for example, found that the richest quintile consumed three to four times the public subsidizes than the poorest quintile. This was mostly because they had far greater access to secondary and tertiary hospital care.

But there are a minority of low income countries that have achieved significant improvements in outcomes in an equitable manner. Countries such as Malawi, Ethiopia, Nepal, Bangladesh, and, more recently, India have sought to achieve universal coverage through innovations in the delivery of services, such as expanding the employment and scope of community health workers; utilizing new technologies to allow community workers to safely diagnose and treat the most important causes of newborn and child deaths; and expanding services offered through child health days and conditional cash transfers. When combined with a focus on increasing demand for services and intensive community engagement aimed at changing detrimental social norms and behaviors, reductions in mortality rates have been dramatic and equitable — offering further options in the journey towards achieving universal coverage.

There are good reasons to suspect that giving greater priority to delivering health care to the most excluded may be more cost-effective even if the actual costs are greater. Excluded populations within countries generally have a larger proportion of children than other groups owing to higher fertility rates. As their rates of child mortality are also often considerably higher than those of more affluent groups, their burden of child deaths constitutes a large share of the national total. In excluded populations, a higher proportion of children die of preventable or treatable infectious diseases or conditions that the children of other groups. And most excluded populations have much lower coverage levels of cost-effective interventions with a proven high impact in reducing major childhood diseases and conditions. Consequently, these populations have the greatest scope for gains in survival and development outcomes. Recent modelling and country case-study work suggests that a more pro-equity approach is not only ethically the right thing to do but is also more efficient.

The importance of the papers presented in this series cannot be overstated. All of the Population Health Implementation and Training (PHIT) Partnership teams directly address the challenges of achieving a more equitable and, hence, more effective response to reducing mortality and morbidity. All take as a central premise the World Health Organizations description of a well-functioning health services as one which delivers “effective, safe, quality personal and non-personal health interventions to those that need them, when and where needed, with minimum waste of resources” [1]. To accomplish this, strategies adopted were tailored uniquely to each site. The populations targeted are in rural populations, which are generally disadvantaged with respect to urban settings. For example, the team in Zambia uses clinical quality improvement as its centerpiece, relying on a robust community outreach component to build community demand for services. Tanzania places community outreach at its centerpiece, deploying a full-time salaried community-based health worker who delivers curative, as well as service-delivery activities. The Rwandan team has combined infrastructure strengthening, quality improvement, and community health workers, while Ghana and Mozambique emphasize leadership and management supported by high-quality health and budget information, as the lead strategy to bolster service delivery.

Because the benefits of health systems strengthening may be unevenly distributed across a population, all of the teams will collect data that permits use of an equity lens to track the process and benefits to the population of health systems strengthening. The equity measure adopted relies on creation of wealth quintiles based on the list of assets included in the most recent national survey in their respective countries as a basis for reporting socioeconomic data. Conceptually, building stronger health systems is a pro-equity strategy. There is growing interest in revisiting the community health worker as a formal member of the health workforce.

All of the PHIT teams, with the exception of Mozambique, incorporate a community-based component. A community-based cadre may help increase health promoting actions at household level, such as handwashing or use of insecticide-treated bednets. Household members may be encouraged to increase their use of health facilities or participate in outreach points. For example, a community-based worker could promote childhood vaccination thereby building demand for health services. In addition, a community-based worker can assist with follow-up and retention in care, which will help ensure that services have their intended benefit (a key role in the Zambian partnership). In some teams (Ghana, Rwanda, and Tanzania), a trained community cadre is delivering curative services, providing treatment for malaria, childhood pneumonia, and diarrhea. There, the services are brought directly to the household, so that a range of barriers to access are overcome (e.g., geographic, financial, or relational — if, for example, health worker attitudes deter utilization.) The Tanzania team has some preliminary baseline data to suggest that their enhanced access to care may offset the disadvantage of lack of maternal education [2]. In Ghana, the community health officer (CHO) who delivers doorstep services has been documented to be pro-equity, with long distance from facility effectively overcome as a barrier to treatment [3].

It is also possible to speculate about other pro-equity pathways of other health system strengthening approaches. In Ghana, budget planning that is mindful of how health expenditure tracks with the disease burden may reduce investment in so-called “prestige projects,” such as advanced technology, and increase attention to low-cost proven interventions that address preventable illness and mortality. Similarly, improved clinical quality (a focus of PHIT activities in Rwanda and Zambia) may increase health worker morale and patient satisfaction, driving more utilization. The Mozambique team relies on leadership and use of operational research and routine data for decision making, enabling local managers to test innovation and assess what is working and what is not.

While no single model for strengthening a health system will emerge from this effort, across all teams the interventions have several clear themes: assure planning capacity and the ability to track success, deliver quality services, and — with the exception of Mozambique — bring services to people where they live. The field will eagerly await the assessment of population impact of these interventions and the extent to which they narrow the equity gap. Both are necessary to assure that all people receive the benefit of health services that they both need and deserve.

## List of abbreviations used

CHO: Community health officer; MDGs: Millennium Development Goals; PHIT: Population Health Implementation and Training

## Competing interests

The author serves as an Expert Advisor to the Doris Duke Charitable Foundation’s African Health Initiative.

## Declarations

This article has been published as part of *BMC Health Services Research* Volume 13 Supplement 2, 2013: Improving primary health care to achieve population impact: the African Health Initiative. The full contents of the supplement are available online at http://www.biomedcentral.com/bmchealthservres/supplements/13/S2. Publication of this supplement was supported by the African Health Initiative of the Doris Duke Charitable Foundation.
